# Physical Activity Interventions Using Digital Health Interventions for Cancer-Related Fatigue in People With a History of Cancer: Scoping Review

**DOI:** 10.2196/83727

**Published:** 2026-06-26

**Authors:** Yeeun Kim, Ka Ryeong Bae, Ji Hyun Sung, Yun Hee Ko, Sue Kim

**Affiliations:** 1 College of Nursing, Yonsei University, Seoul, Republic of Korea; 2 School of Nursing, University of Texas at Austin, Austin, TX, United States; 3 College of Nursing, Eulji University, Seongnam, Republic of Korea; 4 College of Nursing, Keimyung University, Daegu, Republic of Korea; 5 Mo-Im Kim Nursing Research Institute, College of Nursing, Yonsei University, 50-1, Yonsei-Ro, Seodaemun-gu, Seoul, 03722, Republic of Korea, 82 2-2228-3276

**Keywords:** people with a history of cancer, digital health, exercise, fatigue, neoplasm

## Abstract

**Background:**

Although exercise has been proven effective in alleviating cancer-related fatigue (CRF), traditional face-to-face programs may not be accessible due to physical, temporal, or geographical barriers. Digital health interventions (DHIs) offer scalable alternatives for promoting physical activity; however, evidence synthesizing DHI-based physical activity interventions specifically targeting CRF and their intervention characteristics remains limited.

**Objective:**

This scoping review aimed to map the types of digital health–based physical activity interventions for managing CRF, to summarize the key characteristics of DHI modalities and CRF outcomes, and to identify knowledge gaps for future research.

**Methods:**

A systematic literature search was conducted across 4 databases (PubMed, EMBASE, Cochrane, and PsycINFO) up to December 2025. Inclusion criteria comprised experimental studies involving adults with a history of cancer, digital exercise interventions, a control group, and fatigue outcomes. Screening and data extraction followed the Joanna Briggs Institute Manual for Evidence Synthesis and PRISMA-ScR (Preferred Reporting Items for Systematic Reviews and Meta-Analyses Extension for Scoping Review) standards. The protocol was registered in PROSPERO (International Prospective Register of Systematic Reviews; CRD42022304285). Interventions were classified using the Evidence Standards Framework (ESF).

**Results:**

Thirty-three studies comprising 3443 participants were included, representing 32 interventions. Most were randomized controlled trials (n=30, 91%). Interventions were delivered primarily via mobile apps (11/32, 34%) and wearable devices (8/32, 25%), followed by web-based platforms, videoconferencing, exergaming, and augmented reality. Eighteen interventions (reported in 19 studies) demonstrated statistically significant between-group CRF reductions, predominantly at immediate postintervention assessments. Evidence for sustained benefit beyond 12 months was limited, and only one study evaluated ultra-long-term outcomes, which did not demonstrate maintained improvement. Populations with breast cancer accounted for the largest proportion of participants. Fatigue measurement tools varied substantially, potentially contributing to heterogeneity in effect estimates. Most interventions were classified as ESF tier C, indicating a predominant focus on clinical outcome improvement rather than individual-level self-management and system-level implementation. Wearable device-based interventions showed the highest proportion of significant CRF improvement, whereas mobile app–based interventions reported both significant and nonsignificant results. DHIs lasting more than 12 weeks appeared to be associated with more effective CRF outcomes compared to shorter programs. Direct comparisons between in-person and digital delivery were scarce.

**Conclusions:**

Digital health–based physical activity interventions can reduce CRF in people with a history of cancer, with wearable device and longer-duration interventions showing the most favorable outcomes. However, the current evidence is concentrated in populations with breast cancer, and system-level integration remains unexplored. Future research should prioritize diverse populations with cancer, longer follow-up periods, standardized fatigue measurement, and systematic examination of specific intervention components that contribute to CRF reduction. Ultimately, advancing from individual efficacy trials toward scalable, workflow-integrated digital solutions will be key to sustainable CRF management across diverse oncology settings.

## Introduction

Cancer-related fatigue (CRF) is defined as “a distressing, persistent, subjective sense of physical, emotional, and/or cognitive tiredness or exhaustion related to cancer or cancer treatments that interfere with usual functioning” [1]. CRF is one of the most common symptoms of cancer and its treatment and persists for several years after cancer treatment. The prevalence of overall CRF was 43% among people with a history of cancer, and the prevalence of mild CRF was 71% [2]. Since one-third of people with a history of cancer have experienced affective, cognitive, or physical fatigue, CRF is associated with comorbidities, behavioral and psychological factors, and should be continuously assessed and addressed throughout the entire course of survivor care [3]. CRF has been associated with pain, depression, education level, and physical activity [3,4]. Accordingly, clinical practice guidelines, including those from the National Comprehensive Cancer Network, American Society of Clinical Oncology–Society for Integrative Oncology, and European Society for Medical Oncology, consistently recommend that CRF be routinely screened and that nonpharmacological interventions, particularly exercise, be offered as management strategies [1,5,6].

Exercise is strongly recommended to alleviate CRF as a premier nonpharmacological intervention [5,7,8]. Multiple meta-analyses consistently suggested that exercise interventions significantly reduce CRF and improve the quality of life across various cancer types and treatment phases [7-9]. However, face-to-face exercise programs often encounter barriers related to accessibility, costs, and physical limitations of survivors [10,11]. These barriers have been further amplified for people with a history of cancer living in rural or underserved areas, and the COVID-19 pandemic has further highlighted the need for remote and flexible exercise delivery models [12]. This underscores the importance of exploring ways to deliver exercise interventions more feasibly and consistently throughout the survivorship period.

Digital health interventions (DHIs), defined as interventions delivered through digital technologies, have emerged as a scalable and accessible alternative, overcoming spatial and temporal constraints in cancer care [13,14]. In cancer care, DHIs have been applied across multiple domains, including symptom-focused interventions [15], promotion of physical activity through mobile apps [16], and delivery of psychological support via mobile health platforms [17]. These DHIs have been delivered through various modalities, such as mobile apps, web pages, and wearable devices. These approaches may be particularly useful for extending supportive care beyond conventional in-person settings and supporting survivorship care. Given the established role of exercise in managing CRF, DHIs designed to promote physical activity are relevant for people with a history of cancer. However, it remains understudied which types of digital physical activity interventions are associated with improvements in CRF.

Several existing reviews have examined related topics, but each has notable limitations. Previous systematic reviews and meta-analyses of e-health interventions have focused on physical activity as the primary outcome without examining its effects on CRF [18,19]. Other reviews have been limited to specific cancer types, such as breast cancer, restricting the generalizability of their findings [19-21]. A recent scoping review indicated that the effectiveness of DHIs on fatigue varies considerably depending on the intervention’s type and functionality [22]. However, this review did not provide a granular synthesis of physical activity–specific digital interventions or clarify which components are most consistently associated with the reduction of CRF. To address these gaps, this scoping review specifically focuses on physical activity using DHIs, includes all cancer types, and maps the intervention components in relation to CRF. In addition, given the high degree of heterogeneity in digital platforms, exercise protocols, and fatigue assessment tools, a scoping review is the appropriate methodology for this emerging field [23]. It allows for a comprehensive mapping of the evidence and identifies specific intervention characteristics requiring further rigorous evaluation.

Despite the growing body of evidence supporting DHIs for promoting physical activity in people with a history of cancer, it remains understudied how the specific digital components of physical activity interventions relate to CRF across the diverse cancer population. Therefore, this scoping review aims to map the landscape of DHI-delivered physical activity interventions for managing CRF in people with a history of cancer, to summarize the key characteristics of the DHI modalities, CRF assessment methods, and reported CRF outcomes, and to identify knowledge gaps for future research.

## Methods

### Protocol and Registration

The protocol for this review was registered on PROSPERO (International Prospective Register of Systematic Reviews; registered number CRD42022304285). Although the protocol was initially planned as a systematic review, the study was conducted as a scoping review due to the high degree of heterogeneity in digital platforms, exercise protocols, and fatigue assessment tools in the included studies, which made a scoping review more appropriate for comprehensively mapping the evidence of physical activity DHI types for CRF.

### Study Design

This scoping review selected studies on people with cancer engaging in physical activity DHIs targeting CRF and analyzed the findings. The study adhered to the guidelines of the JBI (Joanna Briggs Institute) Manual for Evidence Synthesis [24] and was reported in accordance with PRISMA-ScR (Preferred Reporting Items for Systematic Reviews and Meta-Analyses extension for Scoping Reviews) guidelines [25] (Checklist 1).

### Eligibility Criteria

The key questions for the study were selected according to the JBI Manual for Evidence Synthesis [24] for population, concept, and context (Textbox 1).

Textbox 1. **Population, concept, context**

- Patient: People with a history of cancer (adults aged 18 years and older since diagnosis with cancer, excluding pediatric patients)
- Concept: exercise/physical activity through a digital health intervention (eg, mobile app, web-based, wearable device, etc) and cancer-related fatigue
- Context: open (no limitation)

The specific inclusion criteria were (1) experimental design studies with a control group, (2) using digital delivery methods, (3) employing a physical activity intervention for patients with a history of cancer, (4) reporting fatigue as an outcome, (5) aged 18 years and older at the time of diagnosis of cancer, and (6) studies published in English.

We excluded studies that did not meet one or more of these criteria, such as studies without a physical activity component, lacking digital delivery, not involving people with a history of cancer, without a fatigue outcome, or that were review or protocol papers. Multimodal interventions that included physical activity were included if fatigue outcomes linked to exercise were presented, whereas they were excluded if it was not possible to determine which intervention component influenced the outcome. Studies that used wearable devices solely for self-monitoring purposes and/or used phone follow-ups without interaction focused on the intervention were also excluded.

### Information Sources

A systematic review of the major databases, such as PubMed, EMBASE, PsycINFO, and Cochrane, was primarily conducted until June 2024 and updated on January 6, 2026, for the period up to December 2025. References from relevant review papers were screened to identify additional eligible studies. The study registries, online resources, contacting authors, and additional information sources were not searched in line with the research purpose.

### Search

The search strategy was reported following the PRISMA-Search (Preferred Reporting Items for Systematic Reviews and Meta-Analyses) [26] (Checklist 2). The search formulae comprised MeSH (Medical Subject Headings) terms and text words, with filtering methods employed to increase specificity based on the characteristics of each database. The search period was not limited for the review, which was based on key questions, and inclusion and exclusion criteria were used to select eligible studies. The search and data collection process was initially consulted with a professional librarian and reviewed and discussed in research team meetings. The research team comprised 2 PhDs, 1 graduate student, and 2 doctorally prepared professors of nursing. The existing search strategy was not reused due to inconsistent digital health keywords. After team discussions, a study-specific strategy was developed. The search terms combined the keywords “cancer,” “digital health,” and “cancer-related fatigue,” and the filters were for English and/or adults in each database (see Multimedia Appendix 1 for a full list of search terms). Two research team members (authors YK and YHK) independently conducted at least 1 search in each database individually to check for literature matches, and the retrieved papers were collected and organized using the EndNote 21 program.

### Selection of Sources of Evidence

The screening of the collected data was conducted using Covidence (Veritas Health Innovation) [27] through more than 10 research team meetings. Duplicate records were removed using EndNote 21 (Clarivate) and Covidence. The titles and abstracts of the initially retrieved papers were reviewed independently, and then the full text of papers that appeared relevant was assessed. Inclusion and exclusion criteria were used to select eligible studies, but in cases of disagreement, the full text was reviewed with the entire team until consensus was reached through comprehensive discussion.

### Data Charting Process

Data charting was conducted using Covidence, with 2 reviewers independently extracting data based on a predefined charting form developed from the study objectives.

The data were organized and categorized into tables according to the analysis framework that was created based on the objectives of the study. Studies were identified, categorized, and analyzed according to general characteristics (author, publication year, country, sample size, cancer type and stage, fatigue scale used, and intervention effectiveness) and intervention characteristics (intervention method and intervention delivery format). The Evidence Standards Framework (ESF) [28] was used to classify the DHI into 3 tiers: tier A targets system services to save health care providers’ time and cost; tier B facilitates health management through communication and information, encompassing functional categories such as health care diaries and promoting good health; tier C involves clinical interventions through informing clinical management, driving clinical management, active monitoring, calculating, diagnosing, and treating conditions. In this study, we categorized as grade A if study outcomes included cost analysis of providers’ time or expenses. If the intervention focused on offering general health information without interaction with experts, it was determined as grade B, and if it focused on personalized information or guidance for self-management or utilized interaction with experts to promote good health and healthy lifestyles, it was categorized as grade C.

### Synthesis of Results

The results were synthesized descriptively to map the range and characteristics of digital health–based physical activity interventions targeting CRF. Studies were grouped and summarized according to study characteristics and intervention features, and frequencies were calculated for delivery modalities, intervention duration, ESF tiers, fatigue measurement tools, and intervention effectiveness.

## Results

### Selection of Sources of Evidence

**Figure 1.**
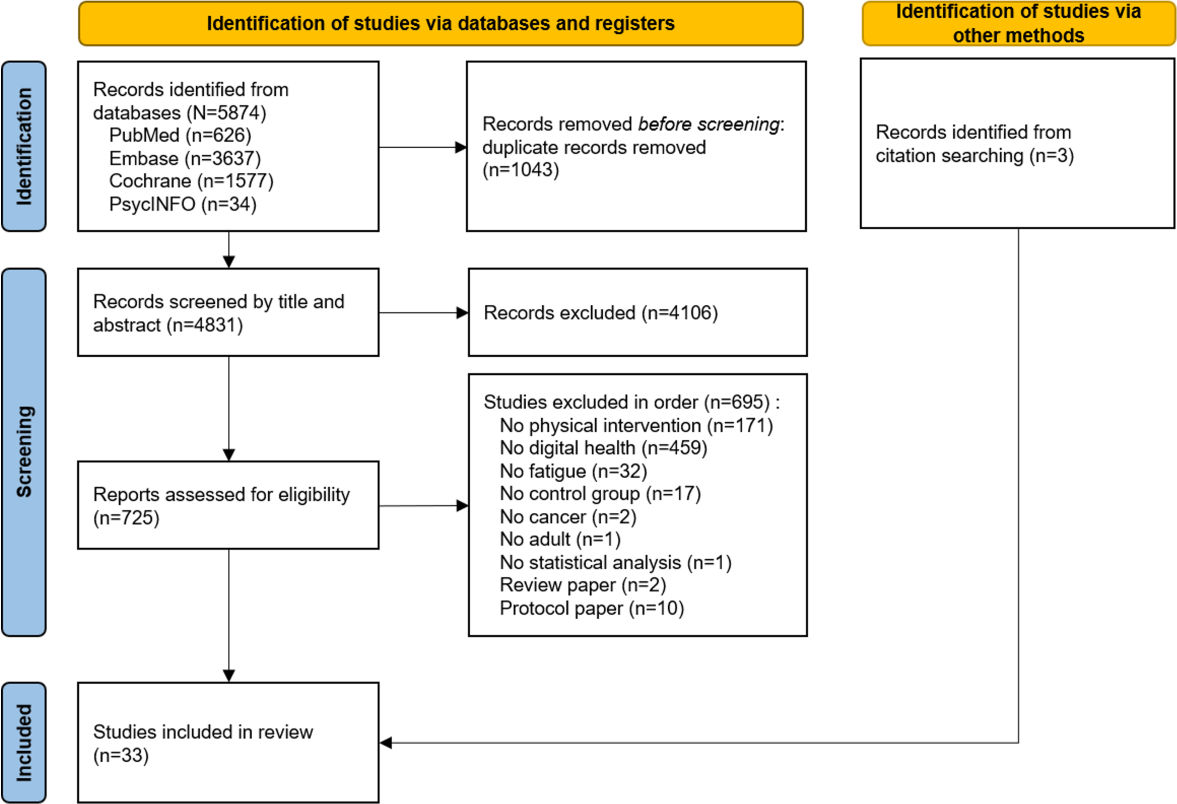
PRISMA (Preferred Reporting Items for Systematic Reviews and Meta-Analyses) 2020 flow diagram of study selection.

A total of 5874 studies were identified based on the search strategy, and 3 studies were identified from citation searching. From this total, 1043 studies were removed due to duplications. Titles and abstracts of the remaining 4831 studies were screened based on the inclusion criteria. Consequently, 725 studies were excluded in the following order: no physical intervention (or unable to determine the effect of physical activity components; n=171), no digital health component in the intervention (n=459), no fatigue results (n=32), no control group (n=17), wrong population (n=2), no statistical analysis (n=1), review paper (n=2), and protocol paper (n=10). Ultimately, 33 studies [29-61] were included in the review (Figure 1).

### Characteristics of Sources of Evidence

The general characteristics of the 33 selected studies are shown in Table 1 and Multimedia Appendix 2. The publication dates ranged from 2015 to 2025, with 26 (79%) studies published after 2020. The United States accounted for the largest number of papers (n=9, 27%), followed, in order, by China (n=5, 15%), Spain (n=4, 12%), Australia (n=3, 9%), the Netherlands (n=3, 9%), and other countries (n=1, 3% each). In terms of study design, the majority (n=30, 91%) were randomized controlled trials (RCTs), and only 3 (9%) were quasi-experimental designs. Most were 2-arm studies (n=29, 88%).

**Table 1.** Publication date, country, study design, and sample characteristics of the 33 selected studies.

Parameter and category	Values, n (%)	References
Publication date		
2019	7 (21)	[29-35]
2020‐2025	26 (79)	[36-61]
Country		
USA	9 (27)	[37,39,41,45,51,54,56,57,59]
China	5 (15)	[34,48,50,55,61]
Spain	4 (12)	[30,42,46,53]
Australia	3 (9)	[36,49,58]
Netherlands	3 (9)	[32,38,44]
Brazil	2 (6)	[33,43]
Belgium	1 (3)	[43]
Canada	1 (3)	[29]
Denmark	1 (3)	[35]
Japan	1 (3)	[40]
Poland	1 (3)	[47]
South Korea	1 (3)	[31]
Taiwan	1 (3)	[60]
Study design		
RCT^a^	30 (91)	[29,30,32,34-36,38-61]
Non-RCT	3 (9)	[31,33,37]
Cancer type		
Single cancer		
Breast cancer	10 (30)	[30,31,36,40,42,46,50,51,53,60]
Hematologic cancer	3 (9)	[45,54,58]
Lung cancer	2 (6)	[52,55]
Others	6 (18)	[34,35,37,47,48,61]
Mixed cancers	5 (15)	[29,32,38,44,49]
All cancer types	7 (21)	[33,39,41,43,56,57,59]
Completion of cancer treatment		
Yes (survivor)	16 (48.5)	[29-31,36,38-40,42,45,46,49,52,53,57-59]
No (patient)	14 (42.4)	[33-35,37,41,43,47,48,50,51,54-56,61]
Mixed sample	3 (9.1)	[32,44,60]
Control group		
Usual care	18 (55)	[29-31,34,35,38,41,42,47,49,50,53,55-60]
Active care	7 (21)	[37,39,40,46,51,52,61]
Waitlist	5 (15)	[32,36,44,45,48]
Others	3 (9)	[33,43,54]

a RCT: randomized controlled trial.

Upon examining key methodological characteristics of the included RCTs, most studies reported randomization and clearly defined control groups, whereas blinding was infrequently reported due to the behavioral nature of the interventions. Sample size and attrition were generally reported, and validated fatigue measures and appropriate statistical analyses were consistently used.

All studies reported aiming to determine the feasibility and suitability of the developed interventions or to evaluate their effectiveness. Two studies [42,44] were conducted to ascertain the long-term effects of the previous studies [30,32].

The total number of participants was 3443, with a range of 20 to 478 participants per study. When classified by cancer types, 21 out of 33 (63%) studies focused on a single type of cancer, of which the most common was breast cancer (10 studies). Five out of 33 (15%) studies included mixed samples of breast, prostate, and/or colorectal cancer, while 7 out of 33 (21%) were nonspecific and recruited all cancers.

Among the 33 studies included, 14 (42%) focused on patients receiving active cancer treatment, whereas 16 (48%) targeted people with a history of cancer who had completed intensive therapy, with less than half noting time since post treatment, which ranged from 1 month [53] to 2 years [45]. Three (9%) studies included participants in both treatment and posttreatment phases.

As presented in Table 1, for the control group, 18 (55%) studies used usual care and 5 (15%) employed a waitlist. Seven (21%) studies employed active care, such as providing control participants with wearable devices without further specification (n=3), allowing access to the mobile app (n=2), and offering a coaching or education program (n=2). The remaining 3 studies were either a noncancer control [33], employed a crossover approach [43], or were factorial designs [54].

### Results of Individual Sources of Evidence

**Figure 2.**
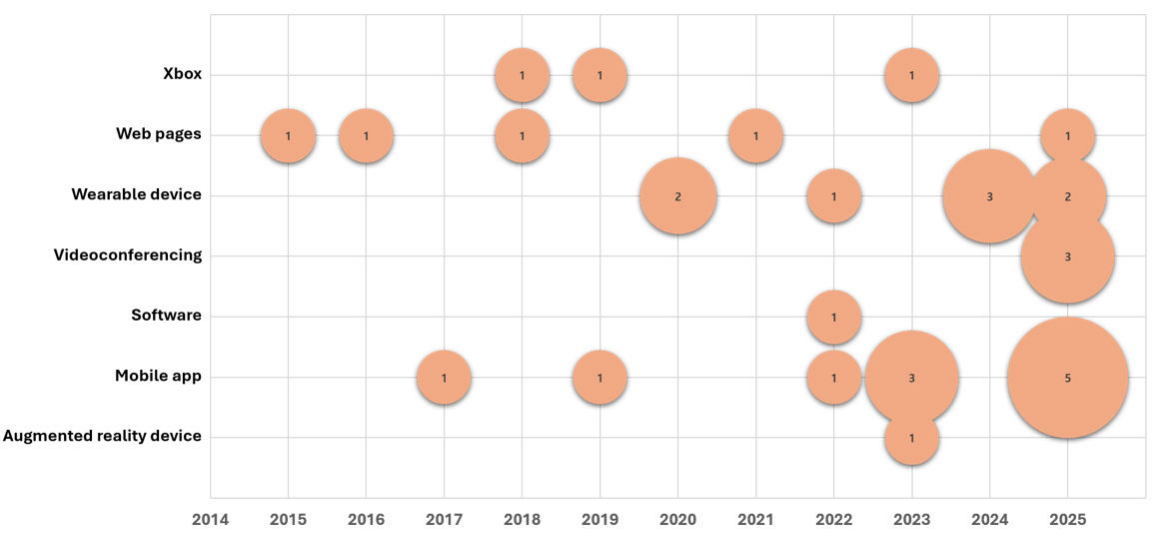
Number of studies by year and intervention modality (n=32 interventions).

Thirty-two interventions were analyzed, excluding studies [42,44] that reported on the same intervention [30,32]. As presented in Table 2 and Multimedia Appendix 3, the most frequent digital therapeutics and delivery method was a mobile app (n=11, 34%), of which most (n=9, 82%) were conducted after 2020, followed by wearable devices (n=8, 25%), such as wristband-type activity monitor devices. Other intervention modalities included a web page (n=5, 16%), videoconferencing (n=3, 9%), Xbox (n=3, 9%), augmented reality (n=1, 3%), and software that could not be classified as strictly mobile app–based or web-based (n=1, 3%). As shown in Figure 2, the number of studies using digital physical activity interventions increased since 2020. Wearable device and videoconferencing interventions emerged after 2020, whereas mobile apps and web page interventions were relatively distributed across the study period.

**Table 2.** Intervention characteristics description of the 32 interventions.

Parameter and category	Values, n (%)	References
Intervention modality		
Mobile app	11 (34)	[31,34,40,45,46,48,55-57,61]
Wearable device	8 (25)	[36,37,39,49-52,54]
Web pages	5 (16)	[29,30,32,38,59]
Videoconferencing	3 (9)	[53,58,60]
Xbox	3 (9)	[33,35,43]
Augmented reality device	1 (3)	[47]
Software	1 (3)	[41]
ESF^a^ grade		
B	2 (6)	[29,35]
C	30 (94)	[30-34,36-41,43,45-61]
Intervention duration		
<12 weeks	9 (28)	[29,30,33,43,45,46,52,58,61]
12 weeks	15 (47)	[31,35-37,39,40,48-51,56,57,59,60]
>12 weeks	8 (25)	[32,34,38,41,47,53-55]

a ESF: Evidence Standards Framework.

The intervention duration ranged from 3 weeks to 6 months, with 12 weeks (n=15, 47%) being the most frequently used period. The detailed intervention characteristics, including physical activity type and digital components, are presented in Multimedia Appendix 3. Most interventions combined digital activity tracking with some form of professional support, such as online coaching, while the specific combination of intervention components varied across studies.

Interventions were graded according to the ESF [28]. Two of the 32 (6%) interventions were grade B, that is, focused on supporting patients’ self-management and wellness; the remaining were grade C, that is, treatment or diagnosis-focused interventions. There was no grade A, which aims to save cost and time for staff, which is likely the nature of the selected studies being focused on testing the efficacy of physical activity for people following cancer diagnosis.

Various fatigue measurements were used in assessments (Table 3). The most frequently utilized scales were the Multidimensional Fatigue Inventory (MFI) and the European Organisation for Research and Treatment of Cancer Quality of Life Questionnaire C-30 (EORTC QLQ C-30) fatigue subscale, each used in 5 (15%) studies. Three (9%) studies each used one of the following: Functional Assessment of Chronic Illness Therapy-Fatigue Scale, Functional Assessment of Chronic Illness Therapy-Fatigue, Piper Fatigue Scale, Patient Reported Outcomes Measurement Information System, or the Functional Assessment of Cancer Therapy-Fatigue or its fatigue subscale. Two (6%) studies each used either the Brief Fatigue Inventory, the Checklist Individual Strength, or the Chalder Fatigue Scale. Three studies used other instruments such as the Fatigue Symptom Inventory, the Multidimensional Fatigue Symptom Inventory—Short Form, and the Schwartz Cancer Fatigue Scale-6. All studies employed fatigue as the primary variable, with the exception of 4 studies [31,49,53,60], where the primary variable was quality of life, and fatigue was reported as a subscale of EORTC QLQ C-30.

**Table 3.** Description of fatigue measurements and statistical effects on fatigue between groups.

Parameter and category	Values, n (%)	References
Fatigue scale^a^		
MFI^b^	5 (15)	[37,38,48,52,61]
EORTC QLQ C-30^c^	5 (15)	[31,49,53,55,60]
FACIT-Fatigue^d^	3 (9)	[36,45,47]
FACIT-F^e^	3 (9)	[33,43,58]
FACT-F^f^	3 (9)	[29,35,54]
PFS^g^	3 (9)	[30,42,46]
PROMIS^h^	3 (9)	[51,56,57]
BFI^i^	2 (6)	[34,59]
CIS^j^	2 (6)	[32,44]
CSF^k^	2 (6)	[40,50]
Fatigue Symptom Inventory	1 (3)	[39]
MSFI-SF^l^	1 (3)	[55]
SCFS-6^m^	1 (3)	[41]
Statistically significant		
Yes (effective)	19 (58)	[30,32-34,36,37,39-41,43,44,46,50-55,60]
No (nonsignificant)	14 (42)	[29,31,35,38,42,45,47-49,56-59,61]

a As some studies used multiple scales, percentages sum to >100%.

b MFI: Multidimensional Fatigue Inventory.

c EORTC QLQ C-30: European Organisation for Research and Treatment of Cancer Quality of Life Questionnaire C-30.

d FACIT-Fatigue: Functional Assessment of Chronic Illness Therapy-Fatigue Scale.

e FACIT-F: Functional Assessment of Chronic Illness Therapy-Fatigue.

f FACT-F: Functional Assessment of Cancer Therapy-Fatigue.

g PFS: Piper Fatigue Scale.

h PROMIS: Patient Reported Outcomes Measurement Information System.

i BFI: Brief Fatigue Inventory.

j CIS: Checklist Individual Strength.

k CFS: Chalder Fatigue Scale.

l MSFI-SF: Multidimensional Fatigue Symptom Inventory—Short Form.

m SCFS-6: Schwartz Cancer Fatigue Scale-6.

In all studies, fatigue was measured a total of 44 times after baseline. Twenty-nine measurements occurred immediately at the conclusion of the intervention [29-31,33-41,43,45-48]. Four studies included midpoint measurements during the intervention [32-34,47], while 4 studies did not assess fatigue during the immediate postintervention period [32,42,44,58]. Twelve measurements focused on longer-term effects within 12 months after the postintervention period [30,32,36,44,46,49,58,59]. Another study evaluated an ultra-long-term effect 5 years later [42] from the original study [30].

### Efficacy on Fatigue

As presented in Table 3, a total of 19 studies (18 interventions) reported a statistically significant reduction in CRF in the intervention group compared with the control group. Two studies employing the same intervention presented a positive effect on CRF 3 months before the end of the intervention and at 6 months [32,44] and 12 months after the intervention [44]. Building on the original report that showed significant results at the 6-month mark [30], its follow-up study [42] examined outcomes 5 years after the intervention. However, no statistically significant improvement in fatigue was noted at this ultra-long-term assessment. Of the 18 interventions that reduced CRF, 7 (39%) were wearable devices, 4 (22%) were mobile apps, 2 (11%) each employed videoconferencing, web pages, or Xbox, and 1 (6%) used software as the DHI component.

**Figure 3.**
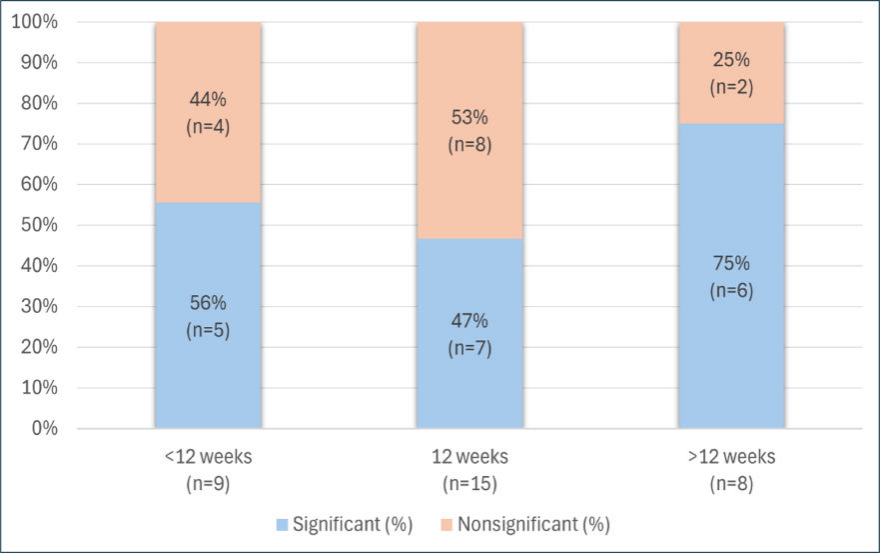
Proportion of studies reporting statistically significant cancer-related fatigue (CRF) improvement by intervention duration (n=32 interventions).

As illustrated in the evidence gap map (Table 4), wearable device interventions showed the highest proportion of statistically significant CRF improvement (7/8, 88% interventions), whereas mobile app interventions showed the lowest proportion of that (4/10, 40%). No studies examined web page interventions for hematologic or lung cancer, and videoconferencing interventions were tested only for individuals with breast cancer and hematologic cancer. As shown in Figure 3, interventions lasting more than 12 weeks presented the highest proportion of significant CRF improvements (6/8, 75%), followed by those lasting less than 12 weeks (5/9, 56%) and those lasting 12 weeks (7/15, 47%).

**Table 4.** Evidence gap map of intervention modality by cancer type and cancer related fatigue across 33 included studies^a^.

Intervention modality	Breast cancer	Hematologic cancer	Lung cancer	Others	Mixed cancers	All cancer types
Mobile app	[31,40,46]	[45]	[55]	[34,48,61]	—^b^	[56,57]
Wearable	[36,50,51]	[54]	[52]	[37]	[49]	[39]
Web pages	[30/42^c^]	—	—	—	[29, 32/44^c^, 38]	[59]
Videoconferencing	[53,60]	[58]	—	—	—	—
Xbox	—	—	—	[35]	—	[33,43]
AR^d^ device	—	—	—	[47]	—	—
Software	—	—	—	—	—	[41]

a The following studies showed statistically significant improvement in CRF between groups: [30,32-34,36,37,39-41,43,44,46,50-55,60].

b Not available.

c References [30] and [32] report the original intervention, and [42] and [44], respectively report its long-term follow-up.

d AR: augmented reality.

## Discussion

### Principal Results

This scoping review mapped the current experimental evidence on physical activity programs delivered via DHIs to address CRF in people with a history of cancer and examined which modality of intervention features tend to improve in CRF. Mobile apps and wearable devices were the most commonly used delivery modalities, followed by web pages, videoconferencing, and exergaming. Twelve-week programs were the most frequently adopted intervention duration, and the evidence has expanded rapidly, with the majority of studies published after 2020, reflecting growing interest in digitally delivered exercise programs for people with a history of cancer. A wide range of fatigue assessment tools was used across the studies, with no single measurement predominating. More than half of the intervention reported statistically significant CRF improvements; however, findings at long-term follow-up were less consistent. Wearable device interventions showed the most consistent decrease in CRF, whereas mobile app interventions demonstrated more mixed findings. Interventions lasting more than 12 weeks were more effective in CRF than those of equal or less than 12 weeks.

Several knowledge gaps were identified. People with a history of breast cancer comprised the largest proportion of the study populations, and evidence for other cancer types, including hematologic or lung cancer, remained limited. Only one study assessed outcomes beyond 5 years, restricting the conclusion regarding the ultra-long-term durability of effects [42]. Furthermore, the high degree of heterogeneity in fatigue assessment tools, control group conditions, and digital delivery modalities made it difficult to directly compare intervention effects across the studies. These gaps highlighted the need for future studies examining diverse populations with cancer, longer follow-up periods, and standardized fatigue assessments to better determine the optimal digital delivery modality and intervention components for CRF reduction.

### Comparison With Prior Work

Previous systematic reviews and meta-analyses have primarily synthesized nondigital exercise interventions or relaxation-based approaches [7,8,62,63]. A meta-analysis [64] reported that eHealth interventions improved pain interference and sleep but not pain severity or fatigue among people with a history of cancer; however, exercise-specific digital interventions and sustainability were not systematically examined. Moreover, recent reviews [18,19,22] have typically aggregated digital interventions across heterogeneous behavioral targets (eg, symptom management, general physical activity, or psychosocial support), without distinguishing exercise-based programs or comparing platform-specific intervention characteristics.

The present review extends prior work by characterizing digital delivery modalities, mapping intervention scope using the ESF [28], and identifying heterogeneity across cancer types, treatment phases, and outcome measurements, thereby informing not only efficacy but also the implementation readiness of digital exercise interventions for CRF. ESF mapping shows that most interventions focus on individual-level support and pay relatively little attention to system-level efficiency and workflow integration. Mobile and wearable-based interventions primarily supported continuous self-monitoring and real-time feedback, which have been identified as core behavior change techniques facilitating awareness and engagement in physical activity promotion [13,14]. In contrast, web-based programs tended to rely more on structured educational content and scheduled interactions, which are typical characteristics of traditional web-based health behavior interventions [65], potentially leading to different engagement patterns and intervention intensity. Most interventions were classified as ESF grade C, with no tier A interventions addressing system-level efficiency [28], indicating opportunities for scalable, implementation-oriented development, and integration into routine oncology service workflows. These modality-specific patterns suggest that the way support is delivered may influence participant engagement and the effective dose of the intervention.

These findings align with a recent meta-analysis, which reported that wearable electronic device system–supported physical activity programs significantly improved the quality of life and physical activity outcomes [66]. The continuous feedback mechanism inherent in wearable devices may enhance self-regulatory processes, thereby facilitating more consistent engagement with physical activity compared to other digital modalities. In contrast, although a previous review reported that mobile app–based interventions enhanced physical activity among people with a history of cancer [18], the present review found that mobile app interventions showed mixed results on CRF. This finding suggests that increased physical activity alone may not be sufficient to improve fatigue outcomes.

Fatigue trajectories after breast cancer treatment are influenced by biobehavioral factors, suggesting that intervention responsiveness may differ across diagnoses [67]. This concentration reflects where physical activity programs delivered via DHIs have been most feasible to test to date, underscoring the need to expand recruitment to more diverse populations with cancer. Given that treatment burden can influence baseline fatigue and responsiveness to exercise-based interventions [7,8,62], heterogeneity in cancer treatment exposure, including surgery, chemotherapy, radiotherapy, immunotherapy, and transplantation, may further constrain intervention outcomes—a consideration when analyzing physical activity DHIs. The mixed follow-up findings suggest that structured support initially improves outcomes, but maintenance may require continued reinforcement or transition-to-maintenance components after the program ends [54]. A recent review on digital health and telehealth in cancer care similarly reported that the evidence for fatigue outcomes was mixed [68]. This suggests that the inconsistent long-term results observed in the present review reflect a broader pattern in the field. Further research is needed to determine how long the CRF benefits of DHIs persist after the completion of intervention and what factors contribute to the maintenance or decrease of these effects over time. Direct comparisons between in-person and digital interventions remain limited, however, as only 2 of our selected studies explicitly compared delivery modalities [57,61]. One study comparing personalized exercise programs delivered in-person versus telehealth found that both interventions improved fatigue, without differences between interventions [57]. The other RCT reported improvement in physical activity with DHIs compared to in-person interventions, but no statistically significant fatigue differences between in-person and DHI modalities [61]. This suggests that whether interventions are delivered digitally or in-person may not determine fatigue outcomes, but the intensity, duration, and content of interventions may be the crucial components of reducing fatigue mechanisms. This highlights a critical evidence gap regarding the comparative effectiveness and implementation efficiency of digital versus in-person interventions for CRF. Given the growing evidence supporting the association between physical activity and CRF in several people with a history of cancer, particularly people with a history of breast and prostate cancer [69], future study should examine whether DHIs on physical activity can produce consistent CRF benefits across more diverse populations with cancer and treatment settings.

### Limitations

This review was limited to English-language publications. Methodological quality was examined using simplified descriptive indicators rather than formal risk-of-bias tools. Although multiple major biomedical databases were searched, the omission of engineering-focused databases (eg, IEEE Xplore) may have limited the identification of some technology-driven interventions.

The inclusion of diverse cancer diagnoses and heterogeneous treatment regimens, including differences in treatment intensity and recovery trajectories, may have influenced baseline fatigue severity and intervention responsiveness, limiting interpretability across populations. People with a history of breast cancer comprised the largest proportion of study populations, limiting generalizability to other cancer types and male populations. In addition, long-term follow-up evidence remains scarce, with only one study assessing outcomes beyond 5 years [42], restricting conclusions regarding the durability of effects. Finally, variability in digital access and literacy may further affect real-world applicability of these findings.

Heterogeneity across the included studies may have influenced the observed results in several ways. First, a wide range of fatigue assessment tools was used across studies, with no single instrument predominating, which limits the direct comparison of CRF outcomes across studies. Second, control conditions varied substantially, ranging from usual care and waitlist controls to active comparators using wearable devices or mobile apps, which may have attenuated detectable intervention effects and limited cross-study comparability. Third, the wide age range of participants, spanning young adults to older survivors, may further contribute to variability in engagement and intervention responsiveness due to differences in digital familiarity and physical capacity [70].

### Conclusions

This scoping review identified several major types of physical activity DHIs for CRF in people with a history of cancer and found that wearable device interventions and longer-duration interventions represented an effective approach for CRF outcomes. Furthermore, as the demand for DHI modalities that actually prompt people with a history of cancer to move may increase in the future, our findings can be used by practitioners as they consider selecting and applying digital health tools to deliver activity-based interventions for alleviating CRF. As the reviewed DHIs were predominantly on patients with breast cancer, clinicians and researchers can respond to the shortcomings identified in this review by actively exploring strategies to engage and include diverse cancer types and underserved populations, while including CRF as a primary outcome.

Overall, this review supports the potential clinical relevance of digital health–based exercise interventions for managing CRF. Beyond individual-level efficacy, the absence of system-level integration (ESF grade A) across the reviewed interventions points to a critical gap between current research and real-world implementation. To enhance real-world impact, future research should prioritize standardized fatigue measurements, broader populations with cancer, and strategies to bridge the digital health literacy gap. Ultimately, shifting the focus from individual efficacy to scalable, workflow-integrated digital solutions will be essential for the sustainable management of CRF in diverse oncology settings.

## Supplementary material

10.2196/83727 Multimedia Appendix 1 Full list of search terms.

10.2196/83727 Multimedia Appendix 2 General characteristics, study aims, and population characteristics of included studies.

10.2196/83727 Multimedia Appendix 3 Summary of intervention characteristics of physical activity and digital health components.

10.2196/83727 Checklist 1 PRISMA ScR checklist.

10.2196/83727 Checklist 2 PRISMA search checklist.

## Abbreviations

CRF: cancer-related fatigue
DHI: digital health intervention
EORTC QLQ C-30: European for Research and Treatment of Cancer Quality of Life Questionnaire C-30
ESF: Evidence Standards Framework
FACIT: Functional Assessment of Chronic Illness Therapy
JBI: Joanna Briggs Institute
MESH: Medical Subject Headings
PRISMA: Preferred Reporting Items for Systematic Reviews and Meta-Analyses
PRISMA-ScR: Preferred Reporting Items for Systematic Reviews and Meta-Analyses extension for Scoping Review
PROSPERO: International Prospective Register of Systematic Reviews
RCTs: randomized controlled trials
