# Supplementary material for: Physical Activity Interventions Using Digital Health Interventions for Cancer-Related Fatigue in People With a History of Cancer: Scoping Review
Source: J Med Internet Res. 2026 Jun 26;28:e83727. doi: 10.2196/83727 (PMC13308905; doi:10.2196/83727)
Supplement: Multimedia Appendix 2 [file jmir-v28-e83727-s002.docx]

**Multimedia Appendix 2.** General characteristics, study aims and population characteristics of included studies (N=33)

| First author (Year) [Ref] | Country | Study design | Sample | Sample size | Fatigue scale | Within group | Between group | Follow up time | Control Group |
| --- | --- | --- | --- | --- | --- | --- | --- | --- | --- |
| Forbes (2015) [1] | Canada | RCT | Localized or metastasized breast, prostate, and colorectal cancer survivors. 96% of participants were disease-free, and the average time since diagnosis was 6.6 years. | I=48, C=47 | FACT-F | Ti0>Ti1, Tc0>Tc1 | Ti0-Ti1 = Tc0-Tc1 | 1: post intervention | Usual care |
| Galiano-Castillo (2016) [2] | Spain | RCT | Stage I, II, IIIA of breast cancer survivors who completed adjuvant therapy. 63% of participants were within 12 months post-surgery. | I=40, C=41 | R-PFS | (-) | Ti1<Tc1, Ti2<Tc2 | 1: post intervention,  2: 6-month follow-up | Usual care |
| Uhm (2017) [3] | South Korea | Quasi-  experimental study | Stage 0-IV of breast cancer survivors who completed primary cancer treatment and ECOG <3. Overall average time since post-treatment were 19.5 months. | I=179, C=177 | EORTC QLQ C-30 fatigue subscale | Ti0>Ti1, Tc0>Tc1 | Ti0-Ti1 = Tc0-Tc1 | 1: post intervention | Usual care |
| Golsteijn (2018) [4] | Netherlands | RCT | Prostate and colorectal cancer patients and survivors who were at least 6 weeks post-surgery | I=249, C=229 | CIS | (-) | Ti0-Ti1 > Tc0-Tc1, Ti0-Ti2 > Tc0-Tc2 | 1: 3 months before the end of intervention,  2: 6 months after intervention | Wait-list |
| Oliveira (2018) [5] | Brazil | Quasi-  experimental study | Stage 0-III of any cancer including breast, gastrointestinal, ovarian, and prostate cancer patients who were undergoing or having previously undergone radiotherapy or chemotherapy. Among cancer group, the mean of cancer diagnosis were 49.8 months. | I=19, C=19 | FACIT-F  (total and fatigue subscale) | Ti0<Ti1, Ti0<Ti2, Tc0=Tc1=Tc2, Ti0<Ti1, Ti0<Ti2, Ti1<Ti2, Tc0=Tc1 | Ti0< Tc0, Ti <Tc (G*E), Ti0<Tc0, Ti1<Tc1, Ti2<Tc2, Ti < Tc(G*E) | 1: 10 sessions complete,  2: post intervention  (20 sessions complete) | Non-cancer control group |
| Lu (2019) [6] | China | RCT | Stage I-III of colorectal cancer patients who underwent surgical resection of gastrointestinal tumors and suffered from CRF | I=45, C=45 | BFI | (-) | Ti0=Tc0, Ti1=Tc1, Ti2<Tc2 | 1: 12 weeks after baseline,  2: post intervention | Usual care |
| Villumsen (2019) [7] | Denmark | RCT | Stage T1c-T3c of prostate cancer patient receiving continuous androgen-deprivation therapy for over 3 months | I=23, C=23 | FACT-F subscale fatigue | (-) | Ti0-Ti1 = Tc0-Tc1 | 1: post intervention | Usual care |
| Vallance (2020) [8] | Australia | RCT | Stage I-III of breast cancer survivors who were inactive and postmenopausal at the time of diagnosis and had completed treatment | I=43, C=40 | FACIT-Fatigue scale | Ti0>Ti1, Ti1=Ti2, Ti1>Ti2, Tc0=Tc1 =Tc2 | Ti0-Ti1 > Tc0-Tc1 | 1: post intervention,  2: 12 weeks after intervention | Wait-list |
| Xiao (2020) [9] | USA | Quasi-  experimental study | Stage II-IV of squamous cell carcinoma in head and neck region patients who were not chronic medical conditions in immune system and were not regularly using immunosuppressive medications | I=12, C=14 | MFI | Ti0=Ti1, Tc0=Tc1 | Ti0=Tc0, Ti1=Tc1, Ti0-Ti1> Tc0-Tc1 | 1: post intervention | Active care (Fitbit) |
| van de Wiel (2021) [10] | Netherlands | RCT | Breast and prostate cancer survivors who had completed primary curative treatment 3–36 months earlier | online I=45, blend I=46, C=46 | MFI | (-) | Toi0-Toi1 = Tc0-Tc1, Tbi0-Tbi1 = Tc0-Tc1 | 1: post intervention | Usual care |
| Johnson (2022) [11] | USA | RCT | Any cancer including lymphoma, brain, and breast cancer survivors who had been from 1-5 years completion of active cancer therapy | I=26, C=23 | Fatigue Symptom Inventory | (-) | Interference: Ti1-Ti0 > Tc1-Tc0 Severity: Ti1-Ti0 = Tc1-Tc0 | 1: post intervention | Active care (Fitbit) |
| Ochi (2022) [12] | Japan | RCT | Stage I-IIA of breast cancer survivors who had surgery within 13 months and were not planned to receive chemotherapy. | I=25, C=25 | CFS | Ti0>Ti1, Tc0<Tc1 | Ti0-Ti1 > Tc0-Tc1 | 1: post intervention | Active care (Fitbit) |
| Wilkie (2022) [13] | USA | RCT | Stage 0-IV and any cancer including breast, head or neck, and sarcoma patients who had been treated | I=142, C=137 | SCFS-6 | (-) | Ti0=Tc0, Ti0-Ti1 > Tc0-Tc1 | 1: post intervention | Usual care |
| Álvarez‑  Salvago (2023) [14] | Spain | RCT | Stage I-IIIA of breast cancer survivors who participated in [2] | I=42, C=38 | PFS | Ti0>Ti1, Tc0>Tc1 | Ti0-Ti1 = Tc0-Tc1 | 1: 5 years after baseline | Usual care |
| da Silva Alves (2023) [15] | Brazil | RCT | Stage 0-III of any cancer including breast, abdominal and pelvic, and gastrointestinal tract cancer patients undergoing chemotherapy | I=28, C=28 | FACIT-F | Ti0<Ti1, Tc0>Tc1 | Ti1-Ti0 > Tc1-Tc0 | 1: post intervention | Cross-over |
| Golsteijn (2023) [16] | Netherlands | RCT | Prostate and colorectal cancer patients and survivors who were at least 6 weeks post-surgery [4]. | I=249, C=229 | CIS | Ti1=Ti2, Tc1=Tc2, Ti0>Ti2, Tc0=Ti2 | Ti1<Tc1, Ti2<Tc2 | 1: 6 months after intervention,  2: 12 months after intervention | Wait-list (access to Onco Active) |
| Lee (2023) [17] | USA | RCT | HCT survivors 2 years after clinical remission, who self-reported as pre-frail or frail | I=10, C=10 | FACIT-Fatigue scale | Ti0=Ti1, Tc0=Tc1 | (-) | 1: post intervention | Wait-list |
| Lozano-Lozano (2023) [18] | Spain | RCT | Stage I-IIIA of breast cancer survivors who were overweight or obese and completed adjuvant therapy. 73% of participants were over 12 months post-surgery. | I=40, C=40 | R-PFS | Ti0<Ti1, Ti0<Ti2, Tc0=Tc1, Tc0=Tc2 | Ti0-Ti1 > Tc0-Tc1, Ti0-Ti2 = Tc0-Tc2 | 1: post intervention,  2: 6 months follow up | Active care (mHealth lifestyle application) |
| Pieczy ́nska (2023) [19] | Poland | RCT | Stage III or IV glioma patients who were ECOG 0-2 and planning to undergo radiotherapy. | I=25, C=22 | FACIT-Fatigue scale | Ti0=Ti1=Ti2, Tc0=Tc1=Tc2 | Ti0-Ti1 = Tc0-Tc1, Ti1-Ti2 = Tc1-Tc2, Ti0-Ti2 = Tc0-Tc2 | 1: 30 days (after RT),  2: post intervention | Usual care |
| Wen (2023) [20] | China | RCT | Stage IV to II and below of nasopharyngeal carcinoma patients who prescribed radiotherapy and chemotherapy and were KPS 80-100 and ECOG score 0-1 | I=44, C=44 | MFI | Ti0>Ti1, Tc0>Tc1 | Ti0-Ti1 = Tc0-Tc1 | 1: post intervention | Wait-list |
| Hardcastle (2024) [21] | Australia | RCT | Breast and colorectal cancer survivors who live in non-metropolitan areas and have low levels of physical activity, and who have finished active treatment within 5 years. | I=43, C=44 | EORTC QLQ C-30 fatigue subscale | (-) | Ti=Tc (group X time) | 1: post intervention,  2: 12 weeks after intervention | Usual care |
| Li (2024) [22] | China | RCT | Stage I-III breast cancer patients undergoing chemotherapy | I=21, C=19 | CFS | Ti0=Ti1, Tc0<Tc1 | Ti0=Tc0, Physical, Affective fatigue: Ti1<Tc1, Cognitive fatigue: Ti1=Tc1 | 1: post intervention | Usual care |
| Phillips (2024) [23] | USA | RCT | Metastatic breast cancer patients who reported insufficient physical activity. Average time since initial diagnosis was 6.3 years; average time since metastatic diagnosis was 2.2 years.  55% were undergoing chemotherapy and 53% were undergoing hormone therapy. | I=25, C=24 | PROMIS Short Form fatigue subscale | (-) | Ti0–Ti1 > Tc0–Tc1 | 1: post intervention | Active care (Healthy lifestyle attention: mobile app) |
| Arents (2025) [24] | Belgium | RCT | Stage I-IIIA of non-small cell lung cancer survivors 3-9 months after completing surgery or chemotherapy | ACP=10, MCP=9 | MFI | Ti0>Ti1, Tc0=Tc1 | Ti0-Ti1>Tc0-Tc1 | 1: post intervention | Active care (Manual coaching program) |
| Lavín-Pérez (2025) [25] | Spain | RCT | Luminal or triple-negative breast cancer survivors who had finished chemotherapy or radiotherapy 1-5 months ago | HRV=18, PE=18. C=18 | EORTC QLQ C-30 fatigue subscale | Thrv0<Thrv1, Tpe0<Tpe1, Tc0>Tc1 | Thrv1-Thrv0>Tc1-Tc0, Thrv1-Thrv0>Tpe1-Tpe0, Tpe1-Tpe0>Tc1-Tc0 | 1: post intervention | Usual care |
| Lee (2025) [26] | USA | RCT | Chronic lymphocytic leukemia patients who were untreated or on stable oral therapy for at least three months, and had at least one lifestyle risk such as being overweight or obese, insufficient exercise, or low consumption of fruit and vegetables | Total=31 | FACT-F | (-) | Tc0=Tc1 (Coaching X exercise), Ts0<Ts1 (Self-monitoring X exercise) | 1: post intervention | None (Factorial design) |
| Li (2025) [27] | China | RCT | Early-stage non-small cell lung cancer patients who had completed surgery 1-2 months ago | I=22, C=18 | MSFI-SF, EORTC QLQ C-30 fatigue subscale | (-) | MSFI-SF: Ti1-Ti0=Tc1-Tc0 EORTC QLQ-30 fatigue subscale: Ti0-Ti1<Tc0-Tc1 | 1: post intervention | Usual care |
| Lukkahatai (2025) a [28] | USA | RCT | Solid cancer tumor patients receiving immunotherapy, including metastatic, breast, prostate, and lung cancer | TEHEplus=10, TEHE=10, ACU=10, C=10 | PROMIS-29 fatigue subsacle | Tteheplus0>Tteheplus1, Ttehe0=Ttehe1, Tacu0=Tacu1, Tc0=Tc1 | Tteheplus0-Tteheplus1=Ttehe0-Ttehe1=Tacu0-Tacu1=Tc0-Tc1 | 1: post intervention | Usual care |
| Lukkahatai (2025) b [29] | USA | RCT | Solid cancer tumor survivors who experienced average fatigue and had completed primary treatment, including metastatic, breast, genitourinary, gastrointestinal, and gynecologic cancer | TEHE=38, iHBE=15, C=22 | PROMIS Short Form | T(tehe+ihbe)0=T(tehe+ihbe)1, Tc0=Tc1 | Ttehe0-Ttehe1=Tihbe0-Tihbe1 | 1: post intervention | Usual care |
| Ma (2025) [30] | Australia | RCT | HCT survivors who were at least 6 months postallogeneic or postautologous HCT | I=73, C=66 | FACIT-F | (-) | Ti0-Ti1=Tc0-Tc1, Ti0-Ti2=Tc0-Tc2, Ti0-Ti3=Tc0-Tc3 | 1: 3 months after baseline,  2: 6 months after baseline,  3: 12 months after baseline (post intervention: 6weeks) | Usual care |
| Unick (2025) [31] | USA | RCT | Cancer survivors who had completed treatment within 3-12 months or were on a maintenance treatment and who also reported insufficient physical activity | I=23, C=23 | BFI | (-) | Ti1=Tc1, Ti2=Tc2 | 1: post intervention,  2: 3 months after intervention | Usual care |
| Yang (2025) [32] | Taiwan | RCT | Stage 0-IV breast cancer patients and survivors perceiving physical or psychological complaints after treatments. Participants were 1469 days post-diagnosis and 51% were undergoing treatment. | I=33, C=33 | EORTC QLQ-C-30 fatigue subscale | Ti0=Ti1, Tc0=Tc1 | Ti0-Ti1>Tc0-Tc1 | 1: post intervention | Usual care |
| Yu (2025) [33] | China | RCT | Stage II-III primary liver cancer patients who were expected to undergo hepatectomy. | I=50, C=50 | MFI | (-) | Ti0-Ti1=Tc0-Tc1 | 1: post intervention | Active care (Rehabilitation education group) |

ACU: Acupressure alone; BFI: Brief Fatigue Inventory; C: control group; CIS: Checklist Individual Strength; CFS: Chalder Fatigue Scale; CRF: cancer-related fatigue; ECOG: Eastern Cooperative Oncology Group; EORTC QLQ C-30: European Organisation for Research and Treatment of Cancer Quality of Life Questionnaire C-30; FACIT-F: Functional Assessment of Chronic Illness Therapy-Fatigue; FACT-F: Functional Assessment of Cancer Therapy-Fatigue; FACIT-Fatigue: Functional Assessment of Chronic Illness Therapy-Fatigue Scale; I: intervention group; HCT: hematopoietic cell transplant; HRV: Heart rate variability; iHBE: Personalized Home-Based Exercise Program; KPS: Karnofsky Performance Status; MFI: Multidimensional Fatigue Inventory; MSFI-SF: Multidimensional Fatigue Symptom Inventory—Short Form; PE: Pre‐planned; PFS: Piper Fatigue Scale; PROMIS: Patient Reported Outcomes Measurement Information System; RCT: Randomized controlled trial; R-PFS: Piper Fatigue Scale-revised; SCFS-6: Schwartz Cancer Fatigue Scale-6; TEHE: Technology-Enhanced Home Exercise; TEHEplus: Technology-Enhanced Home Exercise plus Acupressure

**References**

1. Forbes CC, Blanchard CM, Mummery WK, Courneya KS. Feasibility and Preliminary Efficacy of an Online Intervention to Increase Physical Activity in Nova Scotian Cancer Survivors: A Randomized Controlled Trial. JMIR Cancer 2015;1(2):e12. [doi:10.2196/cancer.4586] [PMID:28410166]

2. Galiano-Castillo N, Cantarero-Villanueva I, Fernandez-Lao C, et al. Telehealth system: A randomized controlled trial evaluating the impact of an internet-based exercise intervention on quality of life, pain, muscle strength, and fatigue in breast cancer survivors. Cancer 2016;122(20):3166-3174. [doi:10.1002/cncr.30172] [PMID:27332968]

3. Uhm KE, Yoo JS, Chung SH, et al. Effects of exercise intervention in breast cancer patients: is mobile health (mHealth) with pedometer more effective than conventional program using brochure? Breast Cancer Res Treat 2017;161(3):443-452. [doi:10.1007/s10549-016-4065-8] [PMID:27933450]

4. Golsteijn RHJ, Bolman C, Volders E, Peels DA, de Vries H, Lechner L. Short-term efficacy of a computer-tailored physical activity intervention for prostate and colorectal cancer patients and survivors: a randomized controlled trial. Int J Behav Nutr Phys Act 2018;15(1):106. [doi:10.1186/s12966-018-0734-9] [PMID:30376857]

5. Oliveira PF, Iunes DH, Alves RS, Carvalho JM, Menezes FS, Carvalho LC. Effects of Exergaming in Cancer Related Fatigue in the Quality of Life and Electromyography of the Middle Deltoid of People with Cancer in Treatment: A Controlled Trial. Asian Pac J Cancer Prev 2018;19(9):2591-2597. [doi:10.22034/APJCP.2018.19.9.2591] [PMID:30256065]

6. Lu Y, Qu HQ, Chen FY, et al. Effect of Baduanjin Qigong Exercise on Cancer-Related Fatigue in Patients with Colorectal Cancer Undergoing Chemotherapy: A Randomized Controlled Trial. Oncol Res Treat 2019;42(9):431-439. [doi:10.1159/000501127] [PMID:31266043]

7. Villumsen BR, Jorgensen MG, Frystyk J, Hordam B, Borre M. Home-based 'exergaming' was safe and significantly improved 6-min walking distance in patients with prostate cancer: a single-blinded randomised controlled trial. BJU Int 2019;124(4):600-608. [doi:10.1111/bju.14782] [PMID:31012238]

8. Vallance JK, Nguyen NH, Moore MM, et al. Effects of the ACTIVity And TEchnology (ACTIVATE) intervention on health-related quality of life and fatigue outcomes in breast cancer survivors. Psychooncology 2020;29(1):204-211. [doi:10.1002/pon.5298] [PMID:31763746]

9. Xiao C, Beitler JJ, Higgins KA, et al. Pilot study of combined aerobic and resistance exercise on fatigue for patients with head and neck cancer: Inflammatory and epigenetic changes. Brain Behav Immun 2020;88:184-192. [doi:10.1016/j.bbi.2020.04.044] [PMID:32330594]

10. van de Wiel HJ, Stuiver MM, May AM, et al. Effects of and Lessons Learned from an Internet-Based Physical Activity Support Program (with and without Physiotherapist Telephone Counselling) on Physical Activity Levels of Breast and Prostate Cancer Survivors: The PABLO Randomized Controlled Trial. Cancers (Basel) 2021;13(15). [doi:10.3390/cancers13153665] [PMID:34359567]

11. Johnson AM, Baker KS, Haviland MJ, et al. A Pilot Randomized Controlled Trial of a Fitbit- and Facebook-Based Physical Activity Intervention for Young Adult Cancer Survivors. J Adolesc Young Adult Oncol 2022;11(4):379-388. [doi:10.1089/jayao.2021.0056] [PMID:34677081]

12. Ochi E, Tsuji K, Narisawa T, et al. Cardiorespiratory fitness in breast cancer survivors: a randomised controlled trial of home-based smartphone supported high intensity interval training. BMJ Support Palliat Care 2022;12(1):33-37. [doi:10.1136/bmjspcare-2021-003141] [PMID:34389552]

13. Wilkie DJ, Schwartz AL, Liao WC, et al. Reduced Cancer-Related Fatigue after Tablet-Based Exercise Education for Patients. Cancer Control 2022;29:10732748221087054. [doi:10.1177/10732748221087054] [PMID:35414203]

14. Alvarez-Salvago F, Jimenez-Garcia JD, Martinez-Amat A, et al. Does participation in therapeutic exercise programs after finishing oncology treatment still ensure an adequate health status for long-term breast cancer survivors? A >/= 5 years follow-up study. Support Care Cancer 2023;31(6):343. [doi:10.1007/s00520-023-07801-8] [PMID:37199790]

15. da Silva Alves R, de Carvalho JM, Borges JBC, Nogueira DA, Iunes DH, Carvalho LC. Effect of Exergaming on Quality of Life, Fatigue, and Strength and Endurance Muscle in Cancer Patients: A Randomized Crossover Trial. Games Health J 2023;12(5):358-365. [doi:10.1089/g4h.2022.0161] [PMID:37155685]

16. Golsteijn RHJ, Bolman C, Peels DA, Volders E, de Vries H, Lechner L. Long-term efficacy of a computer-tailored physical activity intervention for prostate and colorectal cancer patients and survivors: A randomized controlled trial. J Sport Health Sci 2023;12(6):690-704. [doi:10.1016/j.jshs.2023.08.002] [PMID:37591482]

17. Lee K, Shamunee J, Lindenfeld L, et al. Feasibility of implementing a supervised telehealth exercise intervention in frail survivors of hematopoietic cell transplantation: a pilot randomized trial. BMC Cancer 2023;23(1):390. [doi:10.1186/s12885-023-10884-5] [PMID:37127595]

18. Lozano-Lozano M, Galiano-Castillo N, Gonzalez-Santos A, et al. Effect of mHealth plus occupational therapy on cognitive function, mood and physical function in people after cancer: Secondary analysis of a randomized controlled trial. Ann Phys Rehabil Med 2023;66(2):101681. [doi:10.1016/j.rehab.2022.101681] [PMID:35671976]

19. Pieczyńska A, Zasadzka E, Pilarska A, Procyk D, Adamska K, Hojan K. Rehabilitation Exercises Supported by Monitor-Augmented Reality for Patients with High-Grade Glioma Undergoing Radiotherapy: Results of a Randomized Clinical Trial. J Clin Med 2023;12(21). [doi:10.3390/jcm12216838]

20. Wen L, Chen X, Cui Y, Zhang M, Bai X. Effects of Baduanjin exercise in nasopharyngeal carcinoma patients after chemoradiotherapy: a randomized controlled trial. Support Care Cancer 2023;31(1):79. [doi:10.1007/s00520-022-07548-8] [PMID:36562869]

21. Hardcastle SJ, Leyton-Román M, Maxwell-Smith C, Hince D. Impact of the Promoting Physical Activity in Regional and Remote Cancer Survivors intervention on health-related quality of life in breast and colorectal cancer survivors. Frontiers in Oncology 2024;14. [doi:10.3389/fonc.2024.1368119]

22. Li H, Sang D, Gong L, et al. Improving physical and mental health in women with breast cancer undergoing anthracycline-based chemotherapy through wearable device-based aerobic exercise: A randomized controlled trial. Frontiers in Public Health 2024;12:1451101. [doi:https://doi.org/10.3389/fpubh.2024.1451101] [PMID:3257500754; 2025-33360-001]

23. Phillips SM, Starikovsky J, Solk P, et al. Feasibility and preliminary effects of the Fit2ThriveMB pilot physical activity promotion intervention on physical activity and patient reported outcomes in individuals with metastatic breast cancer. Breast Cancer Res Treat 2024;208(2):391-403. [doi:10.1007/s10549-024-07432-5] [PMID:39014267]

24. Arents E, Haesevoets S, Hermans F, et al. Physical Activity Telecoaching in Post-Surgical NSCLC Patients: A Mixed-Methods Pilot Study Exploring Feasibility, Acceptability and Actual Usage. Cancers 2025;17(17). [doi:10.3390/cancers17172886]

25. Lavín-Pérez AM, Collado-Mateo D, Nieto I, et al. Effects of Individualized High-Intensity Online Concurrent Exercise Guided by Autonomic Modulation on the Mental Health and Quality of Life of Breast Cancer Survivors. Psychooncology 2025;34(12):e70348. [doi:10.1002/pon.70348] [PMID:41339114]

26. Lee CY, Gordon MJ, Markofski MM, et al. Optimization of mHealth behavioral interventions for patients with chronic lymphocytic leukemia: the HEALTH4CLL study. J Cancer Surviv 2025;19(4):1325-1334. [doi:10.1007/s11764-024-01555-w] [PMID:38472612]

27. Li G, Zhou X, Deng J, et al. Digital Therapeutics-Based Cardio-Oncology Rehabilitation for Lung Cancer Survivors: Randomized Controlled Trial. JMIR Mhealth Uhealth 2025;13:e60115. [doi:10.2196/60115] [PMID:39999435]

28. Lukkahatai N, Benjasirisan C, Shen A, et al. Combined technology-enhanced home exercise and acupressure (TEHEplus) program on symptoms among cancer patients receiving immunotherapy: a feasibility study. BMC cancer 2025;25(1):1481. [doi:10.1186/s12885-025-14887-2] [PMID:CN-02911908]

29. Lukkahatai N, Han G, Benjasirisan C, et al. A Comparison of In-Person and Telehealth Personalized Exercise Programs for Cancer Survivors: A Secondary Data Analysis. Cancers 2025;17(15). [doi:10.3390/cancers17152432]

30. Ma DD, Liu Z, Au K, et al. Randomized Controlled Trial of a Virtually Delivered Exercise and Stress Management Program to Improve Physical Performance of Hematopoietic Cell Transplant Survivors. Journal of Clinical Oncology 2025;43(8):949-959. [doi:10.1200/JCO.24.00333]

31. Unick JL, Duffy C, Dizon D, et al. Evaluation of a Translatable Web-Based Intervention for Increasing Physical Activity Among Cancer Survivors: Pilot Randomized Trial. JMIR Cancer 2025;11:e79610. [doi:10.2196/79610] [PMID:41037739]

32. Yang YH, Chao YL, Lin YF, Liu PC, Chang KJ, Hou IC. Home-based remote dance program with biopsychosocial model improves quality of life in breast cancer patients: A randomized controlled trial. J Bodyw Mov Ther 2025;43:376-385. [doi:10.1016/j.jbmt.2025.04.029] [PMID:40483151]

33. Yu K, Yin B, Zhu Y, et al. Efficacy of a Digital Postoperative Rehabilitation Intervention in Patients With Primary Liver Cancer: Randomized Controlled Trial. JMIR Mhealth Uhealth 2025;13:e59228. [doi:10.2196/59228] [PMID:40194311]
