# Supplementary material for: Physical Activity Interventions Using Digital Health Interventions for Cancer-Related Fatigue in People With a History of Cancer: Scoping Review
Source: J Med Internet Res. 2026 Jun 26;28:e83727. doi: 10.2196/83727 (PMC13308905; doi:10.2196/83727)
Supplement: Multimedia Appendix 3 [file jmir-v28-e83727-s003.docx]

**Multimedia Appendix 3.** Summary of intervention characteristics of physical activity and digital health components

| First author (Year) [Ref] | Intervention Name | Physical Activity Type | Digital Therapeutics & Delivery Method | Intervention Description | Duration | Frequency | Session Duration | ESF |
| --- | --- | --- | --- | --- | --- | --- | --- | --- |
| Forbes (2015) [1] | UCAN (UWALK website) | Walking, stair climbing | Web-pages | 9-module behavior change program with PA tracking, video guidance, and weekly email updates | 9 weeks | Self-paced | Self-paced | B |
| Galiano-Castillo (2016) [2] | E-CUIDATE system | Aerobics and resistance training | Web-pages | An online system for remote rehabilitation. It is consists of a public interface, which is the homepage with updated information of breast cancer, and a private interface, which could receive personalized exercise programs | 8 weeks | 3 times/week | 90min | C |
| Uhm (2017) [3] | mHealth with pedometer | Walking | Mobile application: Smart After Care | A mobile health intervention using a pedometer and a developed smartphone application to monitor and provide information on prescribed exercises | 12 weeks | Aerobic exercise:  90-150min/week Resistance exercise: 2 times/week | (-) | C |
| Golsteijn (2018) [4] | OncoActive | General physical activity encouragement | Web-pages | A web-based, computer-tailored PA intervention, providing personalized feedback (BL, 2/3 months) and PA tracking | 4 months | PA advice:  3 time points | (-) | C |
| Oliveira (2018) [5] | Exergaming | Aerobic exercise | Xbox: Your Shape Fitness Evolved | An exergaming protocol using Xbox 360 Kinect included 20 sessions of exergaming, focusing on aerobic exercise | 8-10 weeks | 2-3 times/week  (20 session) | 50 min | C |
| Lu (2019) [6] | Baduanjin exercise | Baduanjin | Mobile application: WeChat | A traditional Chinese medicine-based exercise regimen was given face-to-face during hospitalization, and patients were asked to upload everyday log via the WeChat app, and each video was uploaded by every week | 24 weeks | Hospitalization:  5 times/week Chemotherapy period: upload everyday log | Hospitalization: 20-40 min | C |
| Villumsen (2019) [7] | Home-based exergaming | Resistance and aerobic training | Xbox: Your Shape Fitness Evolved | The program was unsupervised exergaming with Xbox Kinect | 12 weeks | 3 times/week | 60 min | B |
| Vallance (2020) [8] | ACTIVATE | General PA including walking | Wearable Device: Garmin Vivofit 2 | A wearable technology-based intervention. Participants received behavioral goal setting and five telephone coaching sessions, and wearable technology activity monitoring | 12 weeks | Self-monitored and  5 phone calls | Self-paced | C |
| Xiao (2020) [9] | Home-based, combined aerobic resistance exercise | Aerobic and resistance training | Wearable Device: Fitbit | The intervention included aerobic exercise and progressive resistance training. The aerobic exercise was tracked with a multi-sensory wristband and based on data research team gave the feedback during weekly clinic visit or by telephone | 12 weeks | Aerobic exercise:  5 times/week Progressive resistance training: 2 times/week | Aerobic: 30 min Progressive resistance training: 20-60 min | C |
| van de Wiel (2021) [10] | IPAS | Aerobic and resistance exercise | Web-pages | An internet-based PA support program (IPAS) for breast and prostate cancer survivors, with and without physiotherapist telephone counseling | 6 months | Monthly | (-) | C |
| Johnson (2022) [11] | Fitbit & Facebook PA Intervention | Walking and general PA | Wearable Device: Fitbit | An intervention using Fitbit and a Facebook support group to encourage physical activity among young adult cancer survivors | 12 weeks | Daily tracking | 150 min/week | C |
| Ochi (2022) [12] | Habit-B programme | High-intensity interval training | Mobile application | A smartphone-supported home-based high-intensity interval training program | 12 weeks | 3 times/week  (daily tracking) | Varies | C |
| Wilkie (2022) [13] | FatigueUCope | Walking | Software | A tablet-based multimedia education and exercise prescription program for managing cancer-related fatigue | 4 weeks | Self-paced  (start with 4 days/week) | Varies | C |
| Álvarez‑ Salvago (2023) [14] | E-CUIDATE system | Same as Galiano-Castillo (2016) | | | | | | |
| da Silva Alves (2023) [15] | Exergaming | Aerobic exercise | Xbox: Your Shape Fitness Evolved | An Xbox Kinect-based exergaming program for cancer patients undergoing chemotherapy, improving muscle strength and fatigue | 20 sessions | 2-3 times/week | (-) | C |
| Golsteijn (2023) [16] | OncoActive | Same as Golsteijn (2018) | | | | | | |
| Lee (2023) [17] | Telehealth exercise | Aerobics and strength training | Mobile application | A supervised telehealth exercise program for frail hematopoietic cell transplant survivors, aiming to improve gait speed and function | 8 weeks | 3 times/week | 30-60 min | C |
| Lozano-Lozano (2023) [18] | BENECA mHealth app | Multimodal rehabilitation exercises | Mobile application | A mobile health application combined with occupational therapy to improve cognitive function, mood, and physical function | 8 weeks | Self-paced and guided | Self-paced | C |
| Pieczy ́nska (2023) [19] | Augmented-reality-based-rehabilitation-exercise | Supervised hospital and home-based exercises | Augmented reality device | A monitor-augmented reality-based rehabilitation exercise program for patients with high-grade glioma undergoing radiotherapy. The study assessed physical fitness, cognitive function, fatigue, and mood | 30 days in hospital and 3 months at home | 5 times/week | 60 min | C |
| Wen (2023) [20] | Baduanjin | Baduanjin | Mobile application | A Baduanjin Qigong exercise program for nasopharyngeal carcinoma patients post-chemoradiotherapy, focusing on improving quality of life, reducing fatigue, and alleviating complications | 12 weeks | 5 days/week,  40 min/day | 40 min | C |
| Hardcastle (2024) [21] | PPARCS | Moderate-to-vigorous PA | Wearable device: Fitbit | Monitor physical activity with Fitbit, and provide goal setting, action plans, problem solving, and feedback through phone coaching. | 12 weeks | 4-6 sessions | 30-60 mim | C |
| Li (2024) [22] | Wearable device-based aerobic exercise | Aerobic exercise | Wearable device | The program begins with the start of the first cycle of chemotherapy, monitoring via the application and a wearable device, while progressively implementing individualized exercise based on the patient's condition. Each session consists of a warm-up, the main aerobic exercise, and flexibility training. | 12 weeks | 3 days/week | 20-45 min | C |
| Phillips (2024) [23] | Fit2ThriveMB | Step-goal physical activity | Wearable device | The Fit2ThriveMB app-based program consists of a ‘daily step increase’ initiative, 24-hour Fitbit wear, and weekly coaching calls. It provides personalized step goal options tailored to daily symptoms and reflects the previous day's goal achievement, designed for participants to self-monitor. | 12 weeks | 1 time/week | Varies | C |
| Arents (2025) [24] | Telecoaching intervention: automated coaching program | Step-goal physical activity with wearable device feedback | Wearable device: Fitbit | An automated coaching program used wearable devices and tailored smartphone application delivering step-goal setting and automated feedback, with an initial motivational interview and telecoach-initiated contact for adherence or technical issues. | 8 weeks | Varies | (-) | C |
| Lavín-Pérez (2025) [25] | HRV-guided exercise & Pre- planned exercise | High-intensity interval and strength exercise | Videoconferencing | Real-time online supervised sessions via video call, including warm up, interval training, strength training and cool down. HRV guided: adjusted intensity daily based on HRV.  Pre-planned: fixed intensity. | 16 weeks | 3 days/week | 60-70 min | C |
| Lee (2025) [26] | HEALTH4CLL | Resistance and aerobic exercise | Wearable device: Fitbit | The program provided Fitbit and materials, while applying a total of 16 intervention combinations varying across the following factors: (1) phone vs email coaching, (2) provision vs non-provision of text reminders, (3) resistance and aerobic exercise vs aerobic exercise alone, and (4) daily vs weekly self-monitoring of weight and dietary intake. | 16 weeks | Telephone coaching:  1 time/week | Telephone coaching: 45-60 min | C |
| Li (2025) [27] | Digital Therapeutics–Based Cardio-Oncology Rehabilitation | Resistance and aerobic exercise | Mobile application: Recovery Plus Health app | Users performed personalized resistance and aerobic exercises via the app's videos. When they transmitted heart rate data and self-reported feedback, AI automatically adjusted prescriptions, which were approved and monitored weekly by physicians. | 5 months | 3-5 day/week,  90-150 min/week | Varies | C |
| Lukkahatai (2025) a [28] | TEHE | Personalized exercise recommendation | Mobile application: TEHEplus app | Personalized recommendations are provided weekly. At the end of each week, exercise recommendations are adjusted based on achievement results. | 12 weeks | Weekly feedback | (-) | C |
|  | TEHEplus | Personalized exercise recommendation | Mobile aplication: TEHEplus app | Combined intervention adding acupressure to TEHE, with weekly symptom-based acupoint recommendations. | 12weeks | Weekly feedback | (-) | C |
| Lukkahatai (2025) b [29] | TEHE | Same as Lukkahatai (2025) a | | | | | | |
|  | iHBE | Personalized exercise recommendation | Mobile application | After a total of 3 home visits during the initial 3 weeks, exercise performance is supported through 9 follow-up phone calls. The application provides real-time monitoring and daily reminders. | 12weeks | Weekly follow-up | (-) | C |
| Ma (2025) [30] | Virtually Delivered Exercise and Stress Management Program | Moderate-to-high intensity exercise with aerobic and resistance activity | Videoconferencing | The program consists of 1:1 video coaching, self-directed aerobic and strength training, and mindfulness-based stress management. Following the intervention, three monthly motivational sessions are provided over a three-month period. | 6 weeks | Telehealth coaching:  1 time/week,  Self-activities:  3 times/week | Telehealth coaching: 60 min, Self-activities 30-40 min | C |
| Unick (2025) [31] | Energize! Exercise Program | Moderate-intensity aerobic exercise | Web-pages | Prescribe progressively increasing weekly exercise goals, submit exercise plans, access multimedia lessons, complete assignments, and track workouts online, with weekly automated personalized feedback provided. | 12 weeks | Feedback message, multimedia lessons, homework:  1 time/week | Multimedia lessons: 10-15 min, Homework: less than 10 min | C |
| Yang (2025) [32] | Home-based remote dance program | Dance program | Videoconferencing | A home-based remote dance program that includes improving muscle strength and range of motion, achieving emotional stability through breathing and relaxation, and promoting social interaction through feedback sharing. Sessions follow a warm-up–guided dance–cool-down structure. | 12 weeks | 1 time/week | 60 min | C |
| Yu (2025) [33] | Digital rehabilitation with R plus Health app | Aerobic and resistance exercise, and stretching training | Mobile application: R Plus Health app | Before discharge, set up the app and heart rate monitor, then perform video-based exercises and transmit relevant data to the physician platform after each session, enabling clinicians to tailor the prescribed regimen. | 3 weeks | 3-5 times/week,  90-150 min/week | 30-50 min | C |

ACU: Acupressure alone; ESF: Evidence Standards Framework; HRV: Heart rate variability; iHBE: Personalized Home-Based Exercise Program; IPAS: Internet-based PA support program; PE: Pre‐planned; PFS: Piper Fatigue Scale; TEHE: Technology-Enhanced Home Exercise; TEHEplus: Technology-Enhanced Home Exercise plus Acupressure

**References**

1. Forbes CC, Blanchard CM, Mummery WK, Courneya KS. Feasibility and Preliminary Efficacy of an Online Intervention to Increase Physical Activity in Nova Scotian Cancer Survivors: A Randomized Controlled Trial. JMIR Cancer 2015;1(2):e12. [doi:10.2196/cancer.4586] [PMID:28410166]

2. Galiano-Castillo N, Cantarero-Villanueva I, Fernandez-Lao C, et al. Telehealth system: A randomized controlled trial evaluating the impact of an internet-based exercise intervention on quality of life, pain, muscle strength, and fatigue in breast cancer survivors. Cancer 2016;122(20):3166-3174. [doi:10.1002/cncr.30172] [PMID:27332968]

3. Uhm KE, Yoo JS, Chung SH, et al. Effects of exercise intervention in breast cancer patients: is mobile health (mHealth) with pedometer more effective than conventional program using brochure? Breast Cancer Res Treat 2017;161(3):443-452. [doi:10.1007/s10549-016-4065-8] [PMID:27933450]

4. Golsteijn RHJ, Bolman C, Volders E, Peels DA, de Vries H, Lechner L. Short-term efficacy of a computer-tailored physical activity intervention for prostate and colorectal cancer patients and survivors: a randomized controlled trial. Int J Behav Nutr Phys Act 2018;15(1):106. [doi:10.1186/s12966-018-0734-9] [PMID:30376857]

5. Oliveira PF, Iunes DH, Alves RS, Carvalho JM, Menezes FS, Carvalho LC. Effects of Exergaming in Cancer Related Fatigue in the Quality of Life and Electromyography of the Middle Deltoid of People with Cancer in Treatment: A Controlled Trial. Asian Pac J Cancer Prev 2018;19(9):2591-2597. [doi:10.22034/APJCP.2018.19.9.2591] [PMID:30256065]

6. Lu Y, Qu HQ, Chen FY, et al. Effect of Baduanjin Qigong Exercise on Cancer-Related Fatigue in Patients with Colorectal Cancer Undergoing Chemotherapy: A Randomized Controlled Trial. Oncol Res Treat 2019;42(9):431-439. [doi:10.1159/000501127] [PMID:31266043]

7. Villumsen BR, Jorgensen MG, Frystyk J, Hordam B, Borre M. Home-based 'exergaming' was safe and significantly improved 6-min walking distance in patients with prostate cancer: a single-blinded randomised controlled trial. BJU Int 2019;124(4):600-608. [doi:10.1111/bju.14782] [PMID:31012238]

8. Vallance JK, Nguyen NH, Moore MM, et al. Effects of the ACTIVity And TEchnology (ACTIVATE) intervention on health-related quality of life and fatigue outcomes in breast cancer survivors. Psychooncology 2020;29(1):204-211. [doi:10.1002/pon.5298] [PMID:31763746]

9. Xiao C, Beitler JJ, Higgins KA, et al. Pilot study of combined aerobic and resistance exercise on fatigue for patients with head and neck cancer: Inflammatory and epigenetic changes. Brain Behav Immun 2020;88:184-192. [doi:10.1016/j.bbi.2020.04.044] [PMID:32330594]

10. van de Wiel HJ, Stuiver MM, May AM, et al. Effects of and Lessons Learned from an Internet-Based Physical Activity Support Program (with and without Physiotherapist Telephone Counselling) on Physical Activity Levels of Breast and Prostate Cancer Survivors: The PABLO Randomized Controlled Trial. Cancers (Basel) 2021;13(15). [doi:10.3390/cancers13153665] [PMID:34359567]

11. Johnson AM, Baker KS, Haviland MJ, et al. A Pilot Randomized Controlled Trial of a Fitbit- and Facebook-Based Physical Activity Intervention for Young Adult Cancer Survivors. J Adolesc Young Adult Oncol 2022;11(4):379-388. [doi:10.1089/jayao.2021.0056] [PMID:34677081]

12. Ochi E, Tsuji K, Narisawa T, et al. Cardiorespiratory fitness in breast cancer survivors: a randomised controlled trial of home-based smartphone supported high intensity interval training. BMJ Support Palliat Care 2022;12(1):33-37. [doi:10.1136/bmjspcare-2021-003141] [PMID:34389552]

13. Wilkie DJ, Schwartz AL, Liao WC, et al. Reduced Cancer-Related Fatigue after Tablet-Based Exercise Education for Patients. Cancer Control 2022;29:10732748221087054. [doi:10.1177/10732748221087054] [PMID:35414203]

14. Alvarez-Salvago F, Jimenez-Garcia JD, Martinez-Amat A, et al. Does participation in therapeutic exercise programs after finishing oncology treatment still ensure an adequate health status for long-term breast cancer survivors? A >/= 5 years follow-up study. Support Care Cancer 2023;31(6):343. [doi:10.1007/s00520-023-07801-8] [PMID:37199790]

15. da Silva Alves R, de Carvalho JM, Borges JBC, Nogueira DA, Iunes DH, Carvalho LC. Effect of Exergaming on Quality of Life, Fatigue, and Strength and Endurance Muscle in Cancer Patients: A Randomized Crossover Trial. Games Health J 2023;12(5):358-365. [doi:10.1089/g4h.2022.0161] [PMID:37155685]

16. Golsteijn RHJ, Bolman C, Peels DA, Volders E, de Vries H, Lechner L. Long-term efficacy of a computer-tailored physical activity intervention for prostate and colorectal cancer patients and survivors: A randomized controlled trial. J Sport Health Sci 2023;12(6):690-704. [doi:10.1016/j.jshs.2023.08.002] [PMID:37591482]

17. Lee K, Shamunee J, Lindenfeld L, et al. Feasibility of implementing a supervised telehealth exercise intervention in frail survivors of hematopoietic cell transplantation: a pilot randomized trial. BMC Cancer 2023;23(1):390. [doi:10.1186/s12885-023-10884-5] [PMID:37127595]

18. Lozano-Lozano M, Galiano-Castillo N, Gonzalez-Santos A, et al. Effect of mHealth plus occupational therapy on cognitive function, mood and physical function in people after cancer: Secondary analysis of a randomized controlled trial. Ann Phys Rehabil Med 2023;66(2):101681. [doi:10.1016/j.rehab.2022.101681] [PMID:35671976]

19. Pieczyńska A, Zasadzka E, Pilarska A, Procyk D, Adamska K, Hojan K. Rehabilitation Exercises Supported by Monitor-Augmented Reality for Patients with High-Grade Glioma Undergoing Radiotherapy: Results of a Randomized Clinical Trial. J Clin Med 2023;12(21). [doi:10.3390/jcm12216838]

20. Wen L, Chen X, Cui Y, Zhang M, Bai X. Effects of Baduanjin exercise in nasopharyngeal carcinoma patients after chemoradiotherapy: a randomized controlled trial. Support Care Cancer 2023;31(1):79. [doi:10.1007/s00520-022-07548-8] [PMID:36562869]

21. Hardcastle SJ, Leyton-Román M, Maxwell-Smith C, Hince D. Impact of the Promoting Physical Activity in Regional and Remote Cancer Survivors intervention on health-related quality of life in breast and colorectal cancer survivors. Frontiers in Oncology 2024;14. [doi:10.3389/fonc.2024.1368119]

22. Li H, Sang D, Gong L, et al. Improving physical and mental health in women with breast cancer undergoing anthracycline-based chemotherapy through wearable device-based aerobic exercise: A randomized controlled trial. Frontiers in Public Health 2024;12:1451101. [doi:https://doi.org/10.3389/fpubh.2024.1451101] [PMID:3257500754; 2025-33360-001]

23. Phillips SM, Starikovsky J, Solk P, et al. Feasibility and preliminary effects of the Fit2ThriveMB pilot physical activity promotion intervention on physical activity and patient reported outcomes in individuals with metastatic breast cancer. Breast Cancer Res Treat 2024;208(2):391-403. [doi:10.1007/s10549-024-07432-5] [PMID:39014267]

24. Arents E, Haesevoets S, Hermans F, et al. Physical Activity Telecoaching in Post-Surgical NSCLC Patients: A Mixed-Methods Pilot Study Exploring Feasibility, Acceptability and Actual Usage. Cancers 2025;17(17). [doi:10.3390/cancers17172886]

25. Lavín-Pérez AM, Collado-Mateo D, Nieto I, et al. Effects of Individualized High-Intensity Online Concurrent Exercise Guided by Autonomic Modulation on the Mental Health and Quality of Life of Breast Cancer Survivors. Psychooncology 2025;34(12):e70348. [doi:10.1002/pon.70348] [PMID:41339114]

26. Lee CY, Gordon MJ, Markofski MM, et al. Optimization of mHealth behavioral interventions for patients with chronic lymphocytic leukemia: the HEALTH4CLL study. J Cancer Surviv 2025;19(4):1325-1334. [doi:10.1007/s11764-024-01555-w] [PMID:38472612]

27. Li G, Zhou X, Deng J, et al. Digital Therapeutics-Based Cardio-Oncology Rehabilitation for Lung Cancer Survivors: Randomized Controlled Trial. JMIR Mhealth Uhealth 2025;13:e60115. [doi:10.2196/60115] [PMID:39999435]

28. Lukkahatai N, Benjasirisan C, Shen A, et al. Combined technology-enhanced home exercise and acupressure (TEHEplus) program on symptoms among cancer patients receiving immunotherapy: a feasibility study. BMC cancer 2025;25(1):1481. [doi:10.1186/s12885-025-14887-2] [PMID:CN-02911908]

29. Lukkahatai N, Han G, Benjasirisan C, et al. A Comparison of In-Person and Telehealth Personalized Exercise Programs for Cancer Survivors: A Secondary Data Analysis. Cancers 2025;17(15). [doi:10.3390/cancers17152432]

30. Ma DD, Liu Z, Au K, et al. Randomized Controlled Trial of a Virtually Delivered Exercise and Stress Management Program to Improve Physical Performance of Hematopoietic Cell Transplant Survivors. Journal of Clinical Oncology 2025;43(8):949-959. [doi:10.1200/JCO.24.00333]

31. Unick JL, Duffy C, Dizon D, et al. Evaluation of a Translatable Web-Based Intervention for Increasing Physical Activity Among Cancer Survivors: Pilot Randomized Trial. JMIR Cancer 2025;11:e79610. [doi:10.2196/79610] [PMID:41037739]

32. Yang YH, Chao YL, Lin YF, Liu PC, Chang KJ, Hou IC. Home-based remote dance program with biopsychosocial model improves quality of life in breast cancer patients: A randomized controlled trial. J Bodyw Mov Ther 2025;43:376-385. [doi:10.1016/j.jbmt.2025.04.029] [PMID:40483151]

33. Yu K, Yin B, Zhu Y, et al. Efficacy of a Digital Postoperative Rehabilitation Intervention in Patients With Primary Liver Cancer: Randomized Controlled Trial. JMIR Mhealth Uhealth 2025;13:e59228. [doi:10.2196/59228] [PMID:40194311]
